# Supplementary material for: A shift in glutamine nitrogen metabolism contributes to the malignant progression of cancer
Source: Nat Commun. 2020 Mar 17;11:1320. doi: 10.1038/s41467-020-15136-9 (PMC7078194; doi:10.1038/s41467-020-15136-9)
Supplement: Supplementary file 1 — Supplementary Information [file 41467_2020_15136_MOESM1_ESM.pdf]

Supplementary Information

A shift in glutamine nitrogen metabolism contributes to malignant progression of cancer

Kodama *et al*.

Supplementary Figures

Supplementary Figures

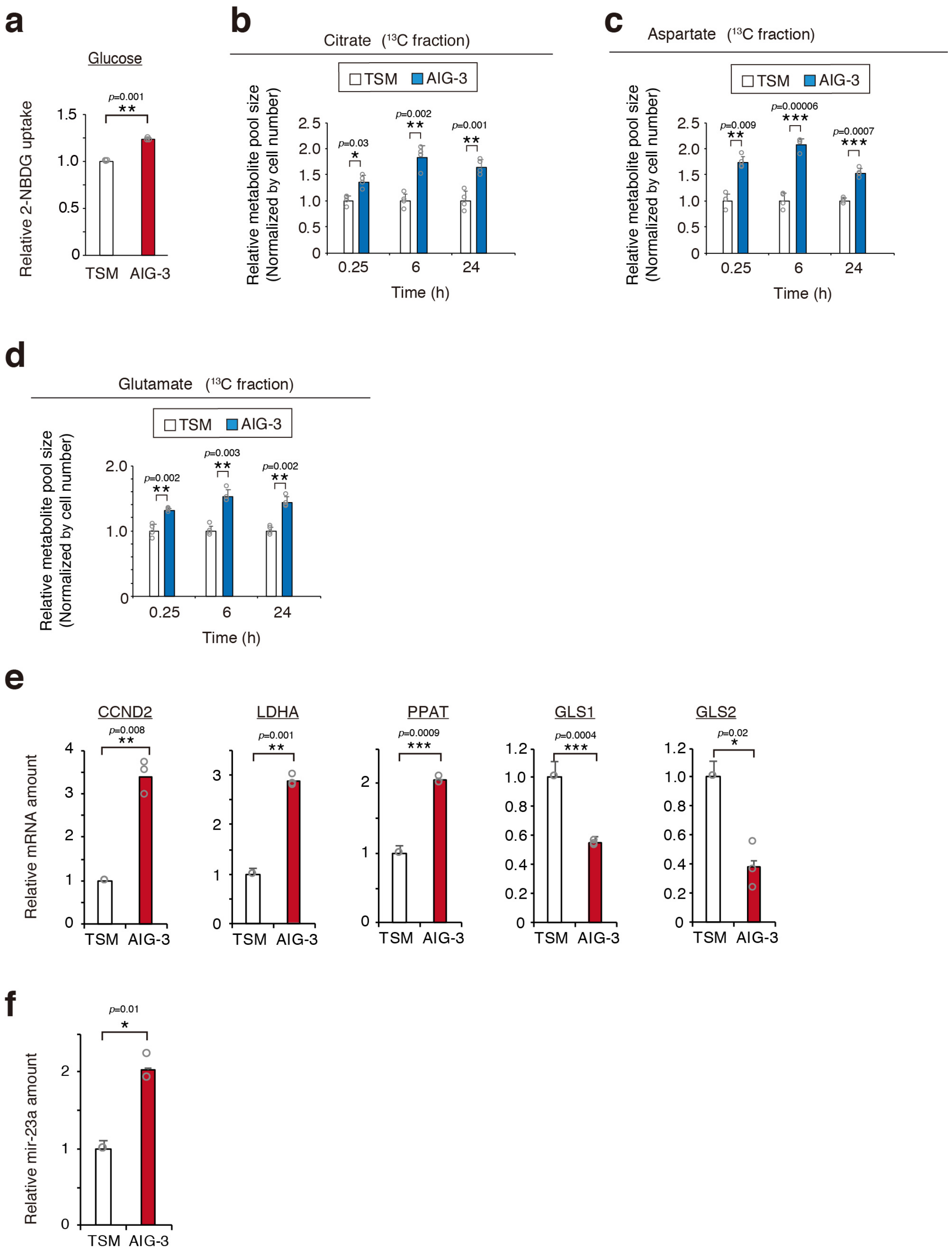

Supplementary Figure 1 | Glucose metabolism and transcriptional activation of c-Myc target genes in AIG-3 cells. a, Glucose uptake in TSM and AIG-3 cells measured with the use of 2-[*N*-(7-nitrobenz-2-oxa-1,3-diazol-4-yl)amino]-2-deoxy-D-glucose (2-NBDG). Measurements were conducted with three biological replicates (n = 3). **b**–**d**, TSM and AIG-3 cells were exposed to 17.5 mM [$^{13}C_6$]glucose in monolayer culture for up to 24 h, after which the metabolite pool size for citrate, aspartate, and glutamate, respectively, was measured by IC-MS or LC-M. All metabolite measurements were conducted with four biological replicates (n = 4). **e**, **f**, Reverse transcription (RT) and real-time polymerase chain reaction (PCR) analysis of mRNAs for c-Myc target genes (n = 3) (**e**) as well as of mir-23a (n = 3) (**f**). In **e** and **f,** measurements were conducted with three biological replicates. All data are means + s.d. *P < 0.05, **P < 0.01, ***P < 0.001 (paired two-tailed Student's t test). Source data are provided as a Source Data file (Source Data 2).

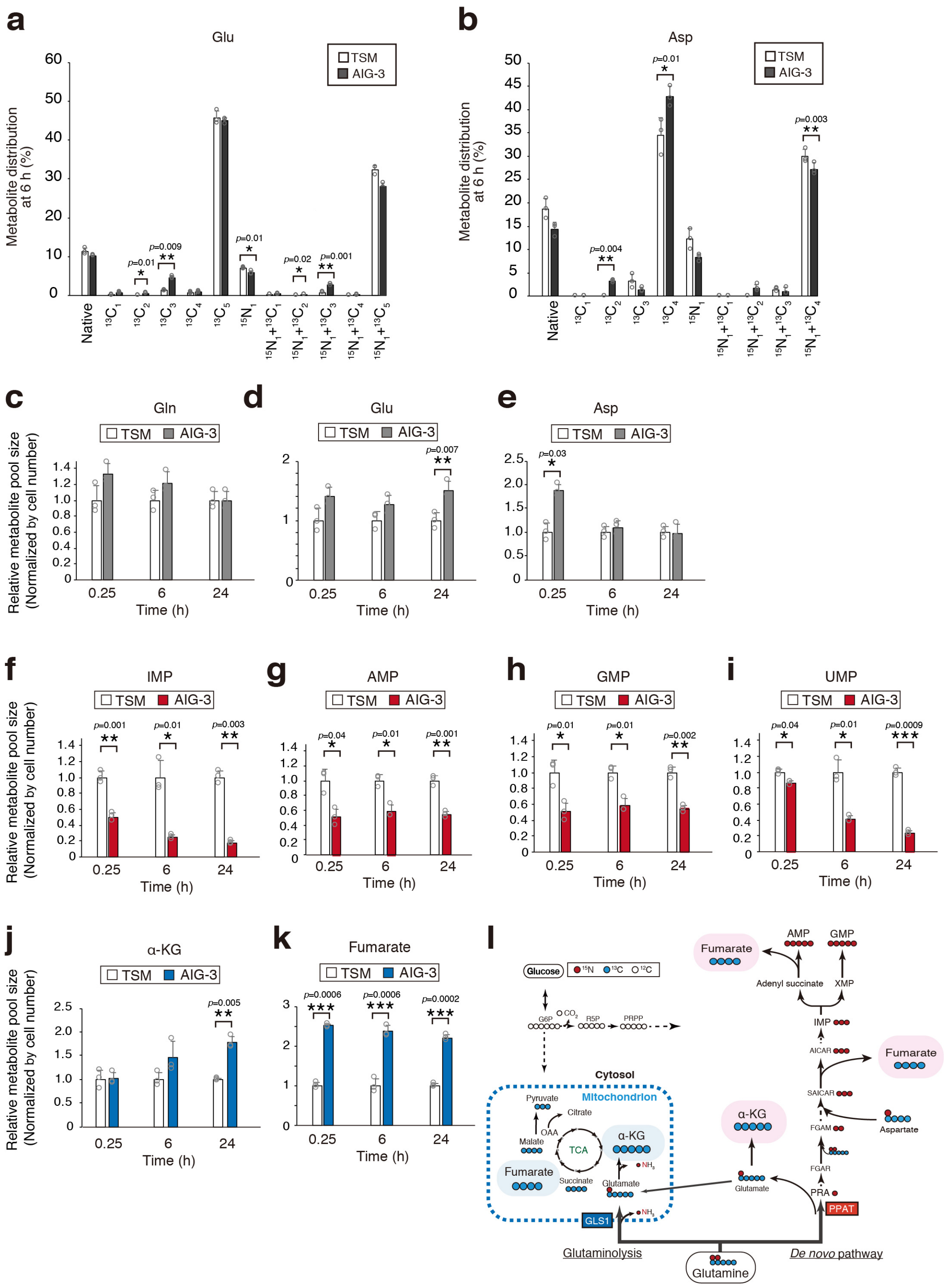

Supplementary Figure 2 | Pool size and isotopologue distribution for metabolites in TSM and AIG-3 cells. a–k, TSM and AIG-3 cells were exposed to 2 mM

[$^{13}C_5$/$^{15}N_2$]glutamine in monolayer culture for up to 24 h, after which the isotopologue distribution for glutamate (**a**) and aspartate (**b**) at 6 h as well as the pool size for the indicated metabolites (**c**–**k**) were measured by IC-MS or LC-M (n = 3). **l**, Schematic representation of [$^{13}C_5$/$^{15}N_2$]glutamine metabolism. α-KG and fumarate are not only metabolites in the glutamine anaplerotic pathway into TCA cycle but also produced by the nucleic acid synthesis pathway and present in the cytosol. All metabolite measurements were conducted with three biological replicates. Data are means + s.d. *P < 0.05, **P < 0.01, ***P < 0.001 (paired two-tailed Student's t test). Source data are provided as a Source Data file (Source Data 2).

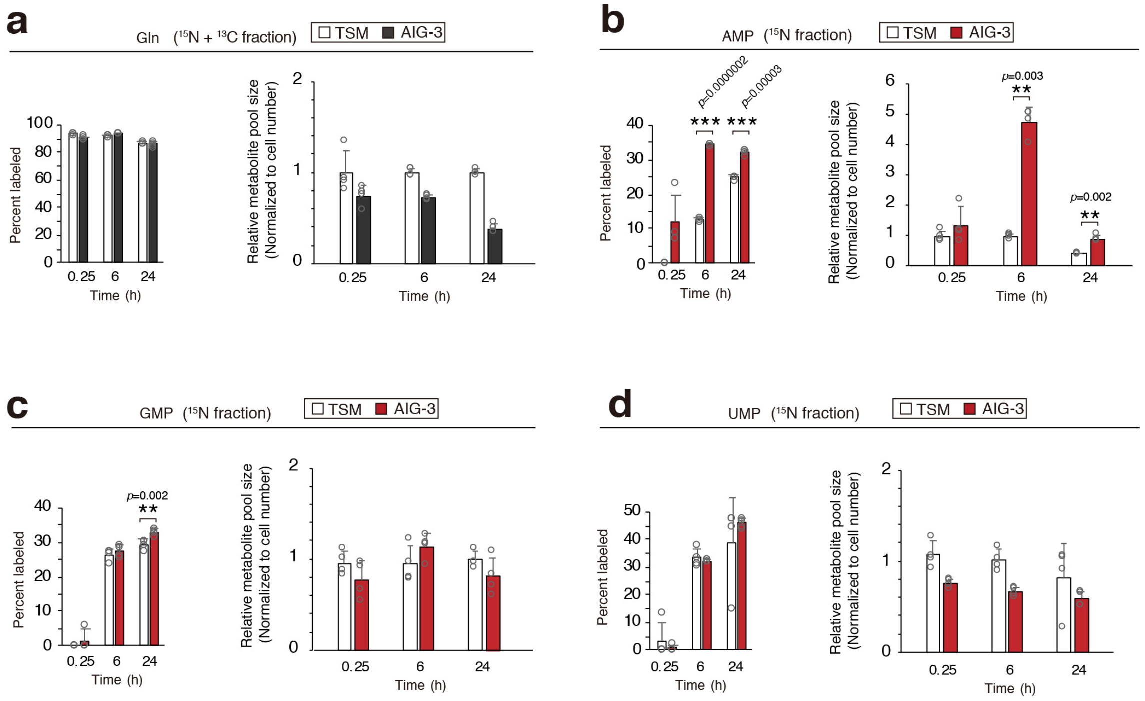

Supplementary Figure 3 | Labeling efficiency and pool size for nucleic acid metabolites in cells cultured in physiological medium. TSM or AIG-3 cells were cultured as monolayers in a physiological medium containing amino acids at the same concentrations as in human plasma for 5 days and were then labeled with 0.6 mM [$^{13}C_5$/$^{15}N_2$] glutamine in the same medium for up to 24 h, after which the labeling efficiency and pool size for glutamine (**a**), AMP (**b**), GMP (**c**), and IMP (d) were determined by IC-MS or LC-M (n = 4). All metabolite measurements were conducted with four biological replicates. Data are means + s.d. *P < 0.05, **P < 0.01, ***P < 0.001 (paired two-tailed Student's t test). Source data are provided as a Source Data file (Source Data 2).

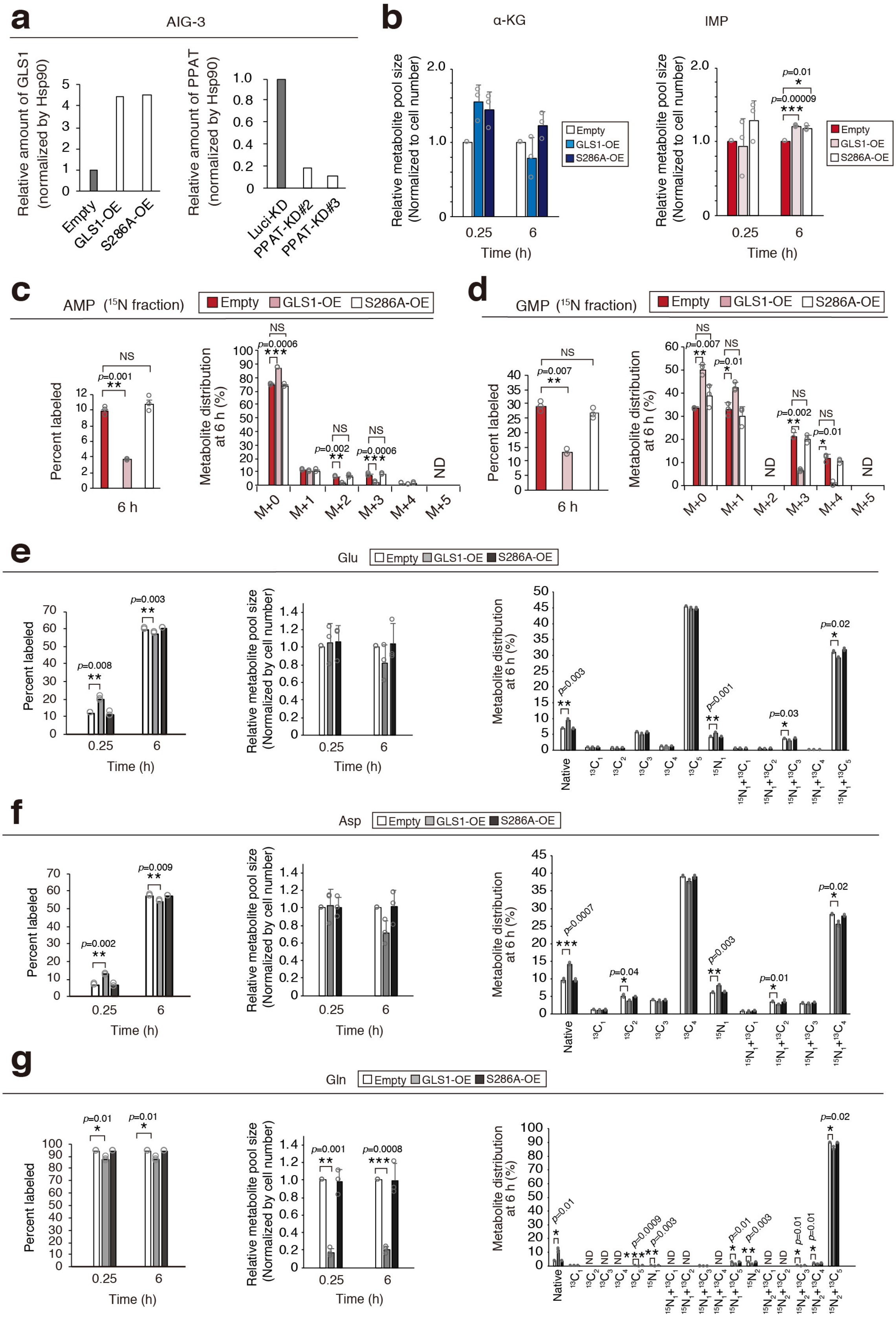
a
AIG-3
Relative amount of GLS1 (normalized by Hsp90)
Empty
GLS1-OE
S286A-OE
Relative amount of PPAT (normalized by Hsp90)
Luci-KD
PPAT-KD#2
PPAT-KD#3
b
α-KG
IMP
Relative metabolite pool size (Normalized to cell number)
Empty
GLS1-OE
S286A-OE
p=0.01
p=0.00009
Time (h)
c
AMP (15N fraction)
Empty
GLS1-OE
S286A-OE
Percent labeled
6 h
Metabolite distribution at 6 h (%)
M+0
M+1
M+2
M+3
M+4
M+5
NS
ND
p=0.001
p=0.0006
p=0.002
d
GMP (15N fraction)
p=0.007
p=0.01
p=0.002
e
Glu
Percent labeled
p=0.008
p=0.003
Relative metabolite pool size (Normalized by cell number)
Metabolite distribution at 6 h (%)
Native
p=0.003
p=0.001
p=0.03
p=0.02
f
Asp
p=0.002
p=0.009
p=0.0007
p=0.04
p=0.003
p=0.01
p=0.02
g
Gln
p=0.01
p=0.001
p=0.0008
p=0.0009
p=0.003
p=0.02
Time (h)

Supplementary Figure 4 | Metabolite pool size, labeling efficiency, and distribution in control and GLS1-overexpressing AIG-3 cells. a, Densitometric analysis of GLS1 and PPAT bands for the immunoblots shown in Figure 4a and 4g, respectively. **b**–**g**, Cells as in Figure 4a were exposed to 2 mM [$^{13}C_5/^{15}N_2$]glutamine in monolayer culture for 0.25 or 6 h, after which the metabolite pool size for ^{13}C-labeled α-KG or ^{15}N-labeled IMP (**b**), the percentage and isotopomer distribution of ^{15}N-labeled AMP (**c**) and GMP (**d**), and the labeling efficiency, metabolite pool size, and isotopologue distribution of glutamate (**e**), aspartate (**f**), and glutamine (**g**) were measured by IC-MS or LC-M analysis. All metabolite measurements were conducted with three biological replicates ($n = 3$). Data in **b** to **g** are means + s.d. *$P < 0.05$, **$P < 0.01$, ***$P < 0.001$ (paired two-tailed Student's t test). Source data are provided as a Source Data file (Source Data 2).

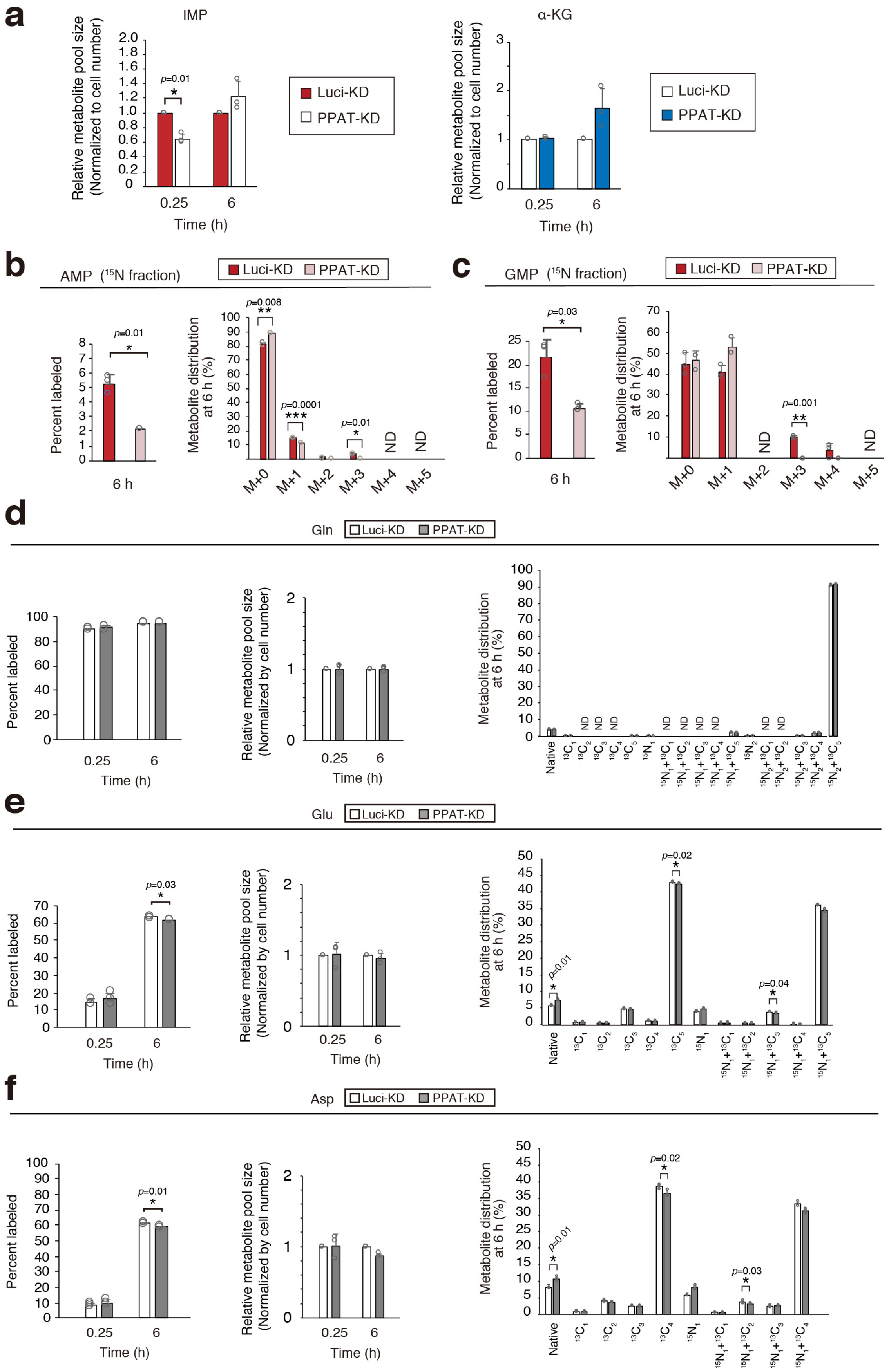
a
IMP
α-KG
Relative metabolite pool size (Normalized to cell number)
p=0.01
Luci-KD
PPAT-KD
Time (h)
b
AMP (15N fraction)
c
GMP (15N fraction)
Percent labeled
Metabolite distribution at 6 h (%)
p=0.008
p=0.0001
p=0.01
p=0.03
p=0.001
ND
6 h
M+0
M+1
M+2
M+3
M+4
M+5
d
Gln
e
Glu
f
Asp
Native
p=0.02
p=0.04
p=0.03
Time (h)

Supplementary Figure 5 | Metabolite pool size, labeling efficiency, and distribution for control and PPAT-depleted AIG-3 cells. a, Cells as in Figure 4g were exposed to 2 mM [$^{13}C_5$/$^{15}N_2$]glutamine in monolayer culture for 0.25 or 6 h, after which the metabolite pool size for ^{15}N-labeled IMP and ^{13}C-labeled α-KG (**a**), the percentage and isotopomer distribution of ^{15}N-labeled AMP (**b**) and GMP (**c**), and the labeling efficiency, metabolite pool size, and isotopologue distribution of glutamine (**d**), glutamate (**e**), and aspartate (**f**) were measured by IC-MS or LC-M analysis. All metabolite measurements were conducted with three biological replicates (n = 3). Data are means + s.d. *P < 0.05, **P < 0.01, ***P < 0.001 (paired two-tailed Student's t test). Source data are provided as a Source Data file (Source Data 2).

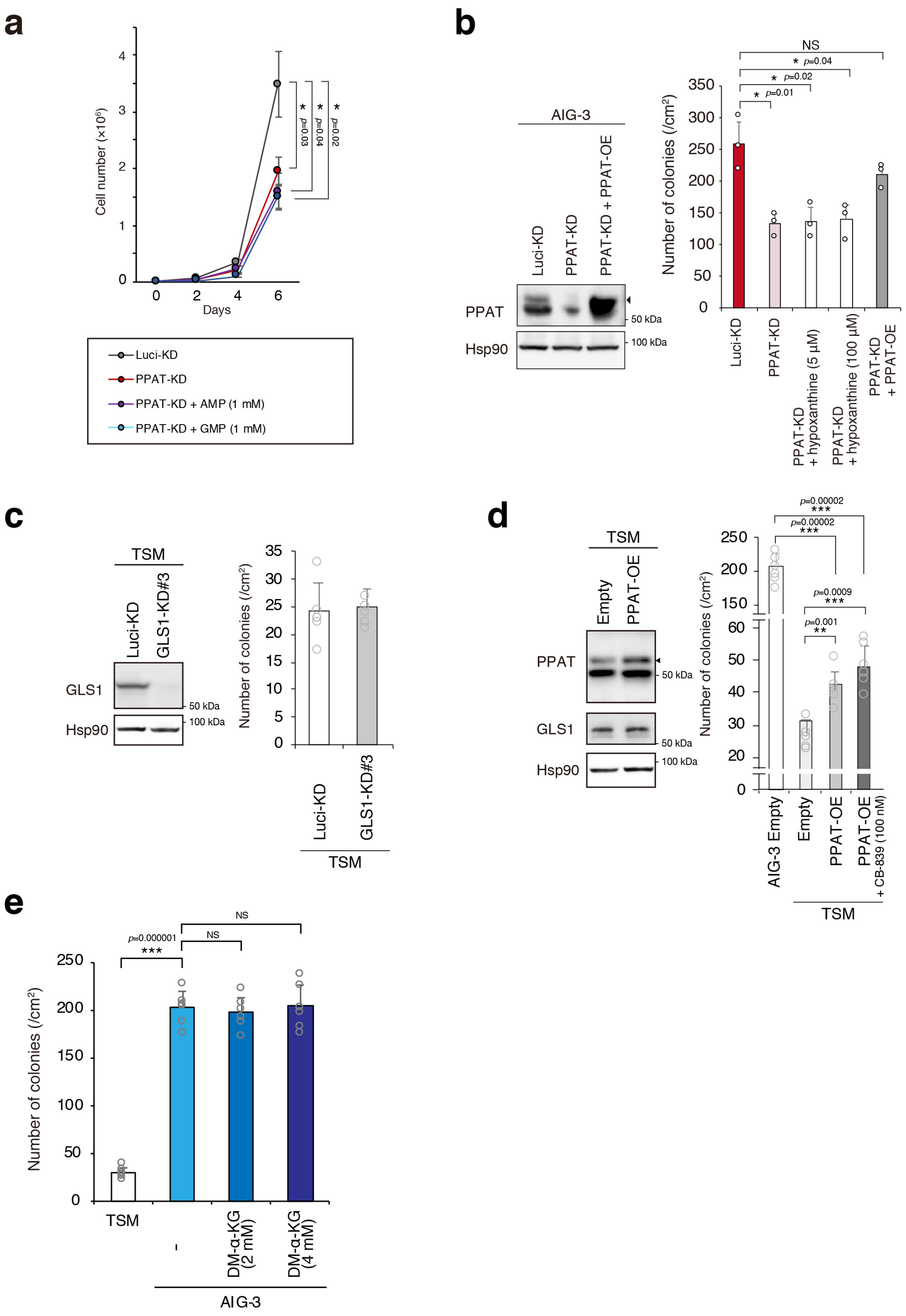
a
Cell number (x10⁶)
Days
p=0.03
p=0.04
p=0.02
Luci-KD
PPAT-KD
PPAT-KD + AMP (1 mM)
PPAT-KD + GMP (1 mM)
b
AIG-3
Luci-KD
PPAT-KD
PPAT-KD + PPAT-OE
PPAT
Hsp90
50 kDa
100 kDa
Number of colonies (/cm²)
NS
p=0.04
p=0.02
p=0.01
PPAT-KD + hypoxanthine (5 µM)
PPAT-KD + hypoxanthine (100 µM)
PPAT-KD + PPAT-OE
c
TSM
GLS1-KD#3
GLS1
d
Empty
PPAT-OE
p=0.00002
p=0.0009
p=0.001
AIG-3 Empty
PPAT-OE + CB-839 (100 nM)
e
p=0.000001
DM-α-KG (2 mM)
DM-α-KG (4 mM)
AIG-3

Supplementary Figure 6 | Up-regulation of PPAT is required but not sufficient for malignant transformation. a, Supplementation of AMP (1 mM) or GMP (1 mM) to PPAT-depleted AIG-3 cells in monolayer culture. All cell count was conducted with three biological replicates (n = 3). **b**, Anchorage-independent growth of AIG-3 cells stably infected with retroviruses encoding luciferase control (Luci-KD) or PPAT (PPAT-KD) shRNAs as well as of PPAT-depleted cells either cultured in the presence of 5 or 100 μM hypoxanthine or infected with a retrovirus for wild-type human PPAT (PPAT-OE). Colony assays were conducted with three biological replicates (n = 3). **c**, Anchorage-independent growth of TSM cells stably infected with retroviruses encoding luciferase control or GLS1 shRNAs (n = 6). **d**, Anchorage-independent growth of AIG-3 or TSM cells infected with an empty retrovirus or of TSM cells stably overexpressing (OE) wild-type PPAT and exposed (or not) to 100 nM CB-839 (n = 6). **e**, Anchorage-independent growth of TSM cells or of AIG-3 cells exposed to 0, 2, or 4 mM dimethyl α-KG (DM-α-KG) (n = 6). In **c** and **d**, colonies were counted in two randomly selected fields in each of three dishes. All data are means + s.d. *P < 0.05, **P < 0.01, ***P < 0.001 (paired two-tailed Student's t test). Source data are provided as a Source Data file (Source Data 2).

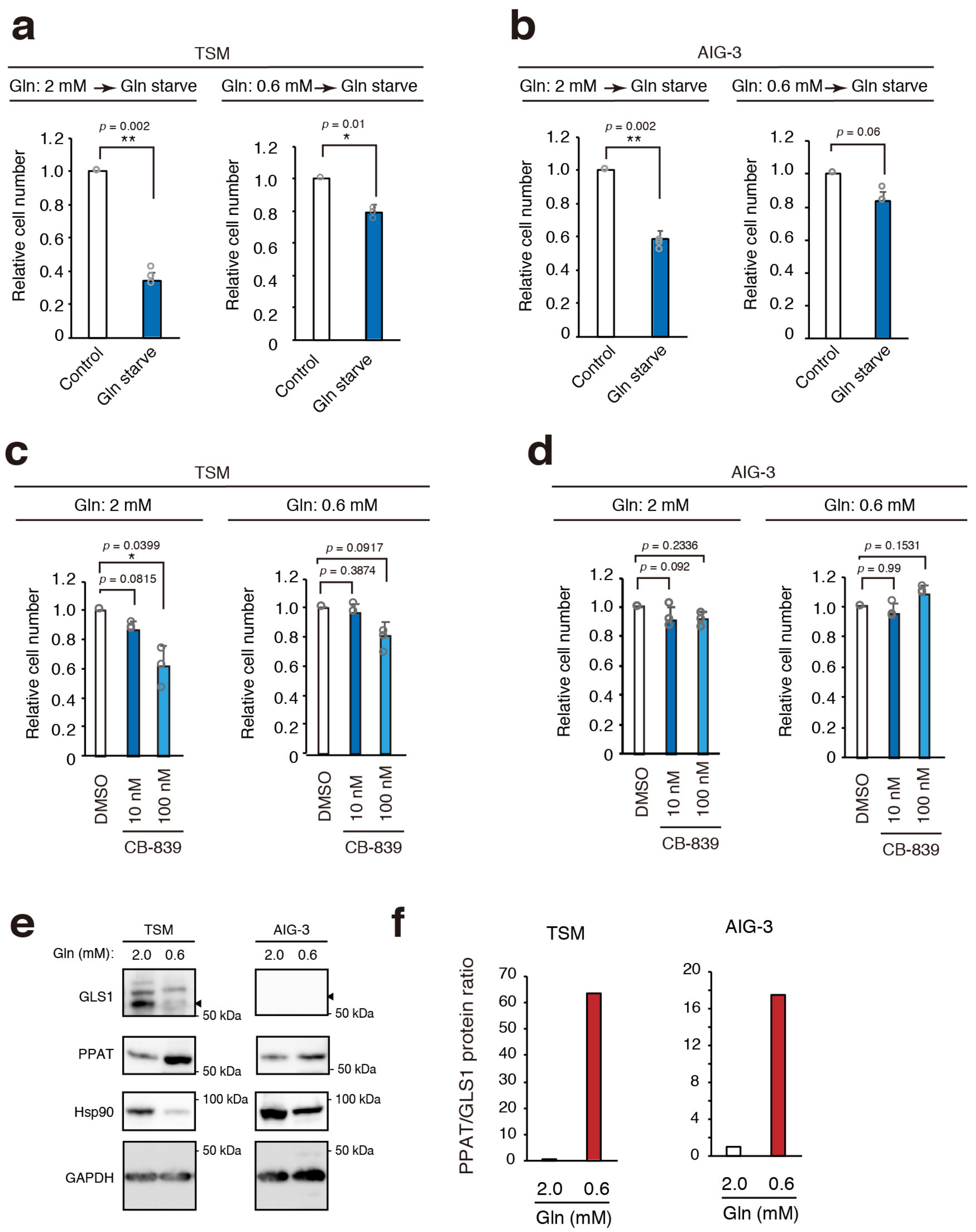

Supplementary Figure 7 | Growth-inhibitory effect of CB-839 depends on the extracellular glutamine concentration. a, **b**, TSM and AIG-3 cells, respectively, were cultured as monolayers either in conventional medium containing 2 mM glutamine or in physiological medium containing amino acids at the same concentrations as in human plasma (including 0.6 mM glutamine), after which the cells were deprived of glutamine

for 48 h and their survival rate determined (*n* = 3). **c**, **d**, Equal numbers (5×10^5) of TSM and AIG-3 cells, respectively, were cultured as monolayers in conventional (2 mM glutamine) or physiological (0.6 mM glutamine) medium and in the presence of vehicle alone (DMSO) or the indicated concentrations of CB-839 for 48 h, after which relative cell number was determined. All cell count was conducted with three biological replicates (*n* = 3). Data in **a** to **d** are means + s.d. *$P < 0.05$, **$P <0.01$ (paired two-tailed Student's *t* test). **e**, **f**, The PPAT/GLS1 ratio in TSM and AIG-3 cells cultured as monolayers in conventional (2 mM glutamine) or physiological (0.6 mM glutamine) medium was determined by immunoblot analysis and densitometry. Both Hsp90 and GAPDH were examined as loading controls. The arrowhead indicates the GLS1 band. All immunoblot analyses were conducted with single time. Source data are provided as a Source Data file (Source Data 2).

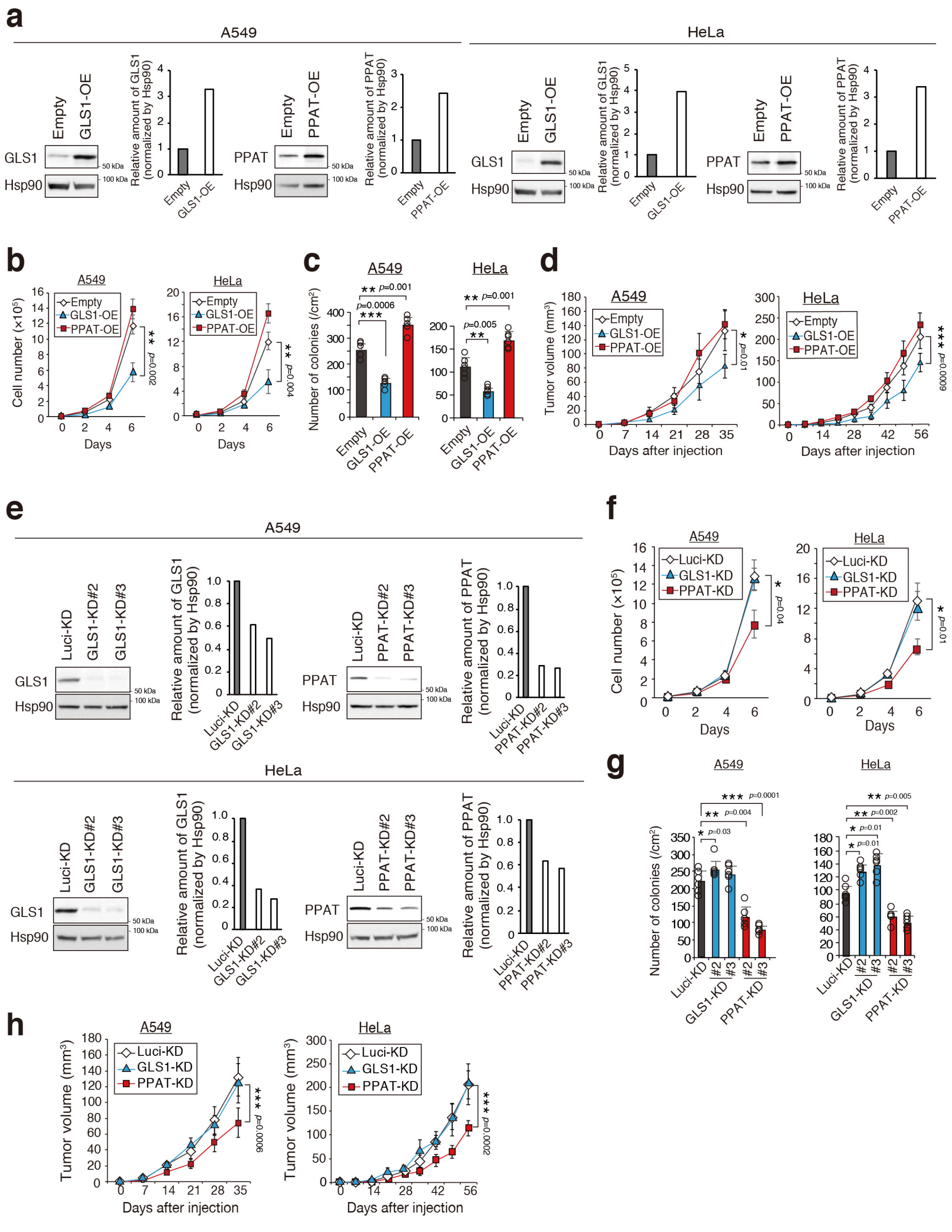

Supplementary Figure 8 | Interventions that reduce the PPAT/GLS1 ratio in human cancer cells suppress tumor growth. a, Immunoblot analysis of GLS1 or PPAT in A549 or HeLa cells stably overexpressing (OE) GLS1 or PPAT or infected with the corresponding empty retrovirus. Immunoblot signals for GLS1 and PPAT were

quantified by densitometry. **b**–**d**, Proliferation rate in 2-D culture (*n* = 3) (**b**), anchorage-independent growth (*n* = 6) (**c**), and tumorigenicity in nude mice (*n* = 7) (**d**) for cells as in **a**. **e**, Immunoblot analysis of GLS1 or PPAT in A549 or HeLa cells stably infected with retroviruses encoding luciferase control (Luci-KD) or two independent GLS1 (GLS1-KD) or PPAT (PPAT-KD) shRNAs. **f**–**h**, Proliferation rate in 2-D culture (*n* = 3) (**f**), anchorage-independent growth (*n* = 6) (**g**), and tumorigenicity in nude mice (*n* = 7) (**h**) for cells as in **e**. All cell count was conducted with three biological replicates. All colonies were counted in two randomly selected fields in each of three dishes. Data in **b** to **d** and in **f** to **h** are means ± s.d. **P* < 0.05, ***P* < 0.01, ****P* < 0.001 (paired two-tailed Student's *t* test). Source data are provided as a Source Data file (Source Data 2).

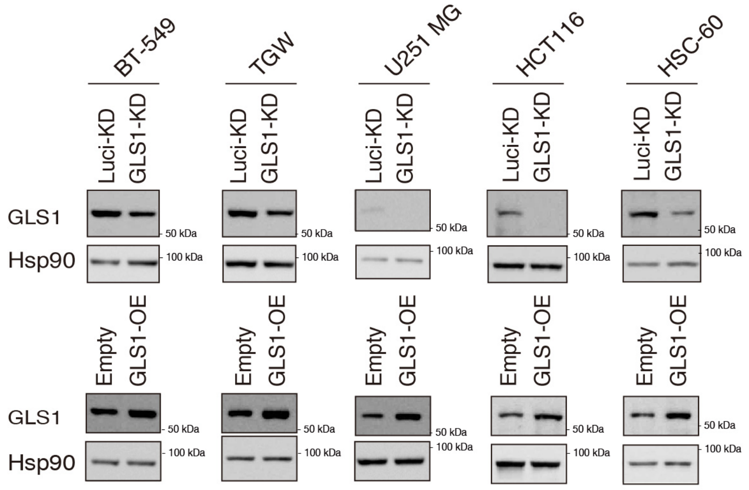

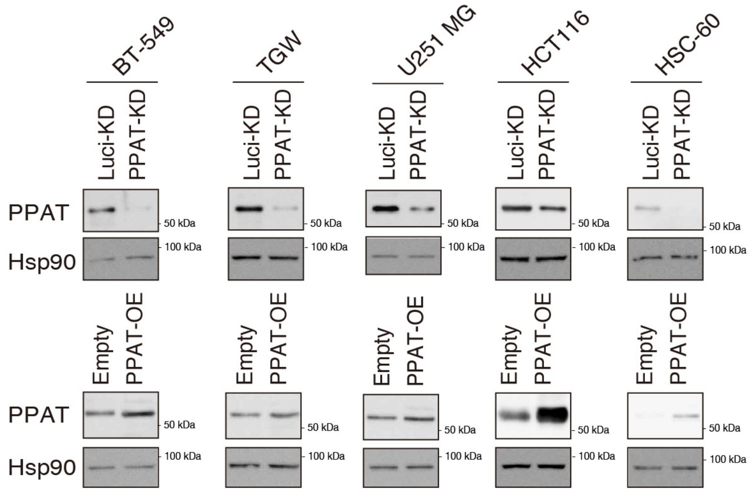

Supplementary Figure 9 | Perturbation of the PPAT/GLS1 ratio in human cancer cell lines. The effects of knockdown (KD) or overexpression (OE) of GLS1 or PPAT by retroviral infection on the abundance of the corresponding proteins were determined by immunoblot analysis. All immunoblot analyses were conducted with single time. Source data are provided as a Source Data file (Source Data 2).

a

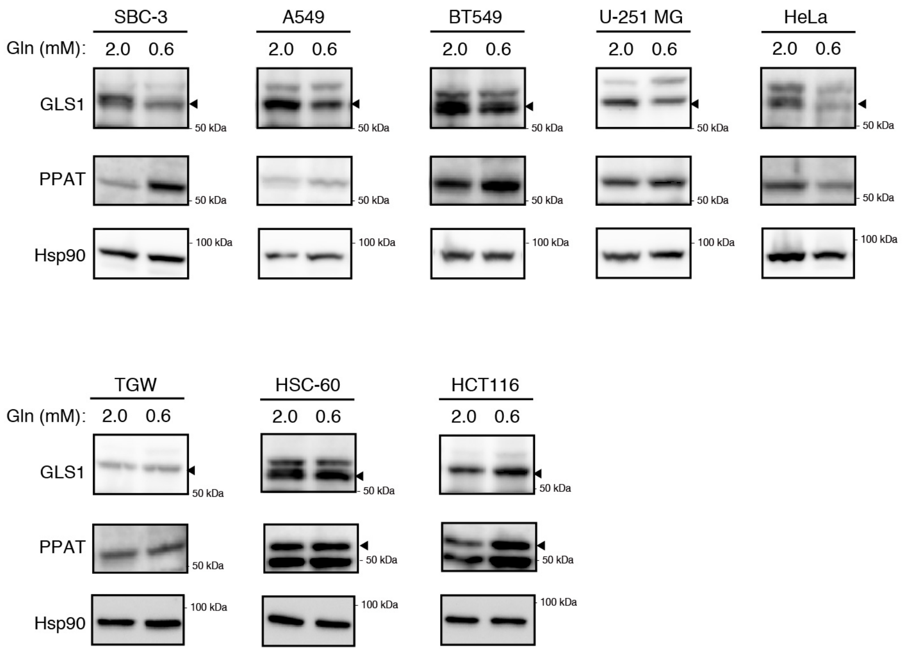

SBC-3
A549
BT549
U-251 MG
HeLa
Gln (mM): 2.0 0.6
GLS1
PPAT
Hsp90
50 kDa
100 kDa
TGW
HSC-60
HCT116

b

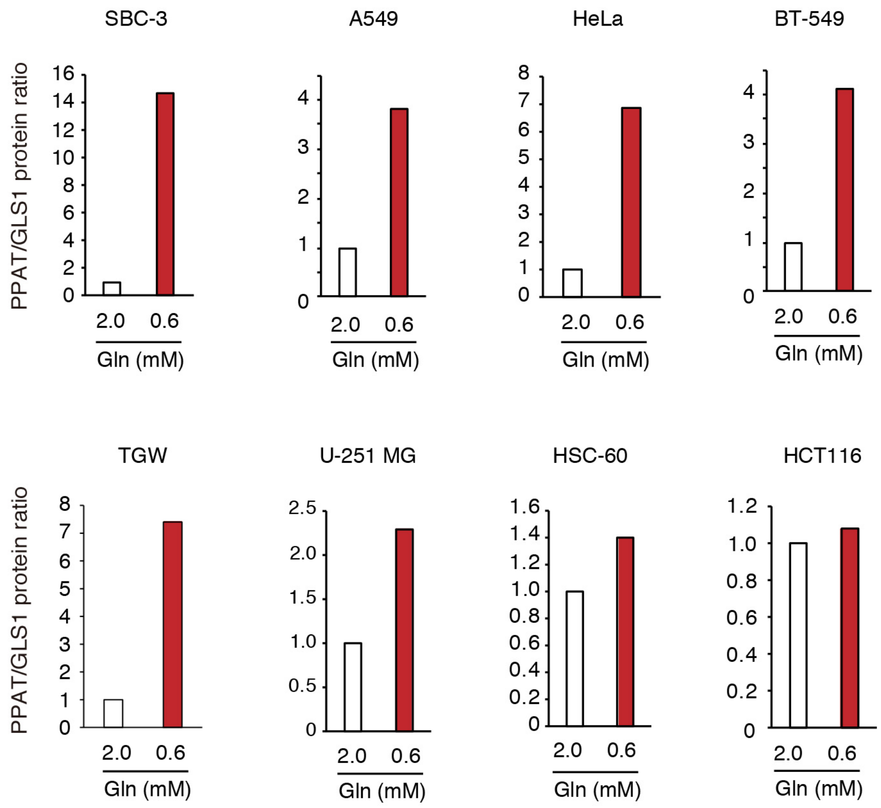

SBC-3
A549
HeLa
BT-549
TGW
U-251 MG
HSC-60
HCT116
PPAT/GLS1 protein ratio
2.0 0.6
Gln (mM)

Supplementary Figure 10 | The PPAT/GLS1 ratio is increased in human cancer cell lines cultured in physiological medium (0.6 mM glutanine) compared with conventional medium (2 mM glutamine). a, Immunoblot analysis of GLS1 and PPAT in human cancer cell lines cultured as monolayers in conventional medium (containing 2 mM glutamine) or in a physiological medium containing amino acids at the same concentrations as in human plasma (including 0.6 mM glutamine). Arrowheads indicate specific immunoreactive bands. **b**, The PPAT/GLS1 ratio determined by densitometry from the immunoblots in **a**. All immunoblot analyses were conducted with single time. Source data are provided as a Source Data file (Source Data 2).

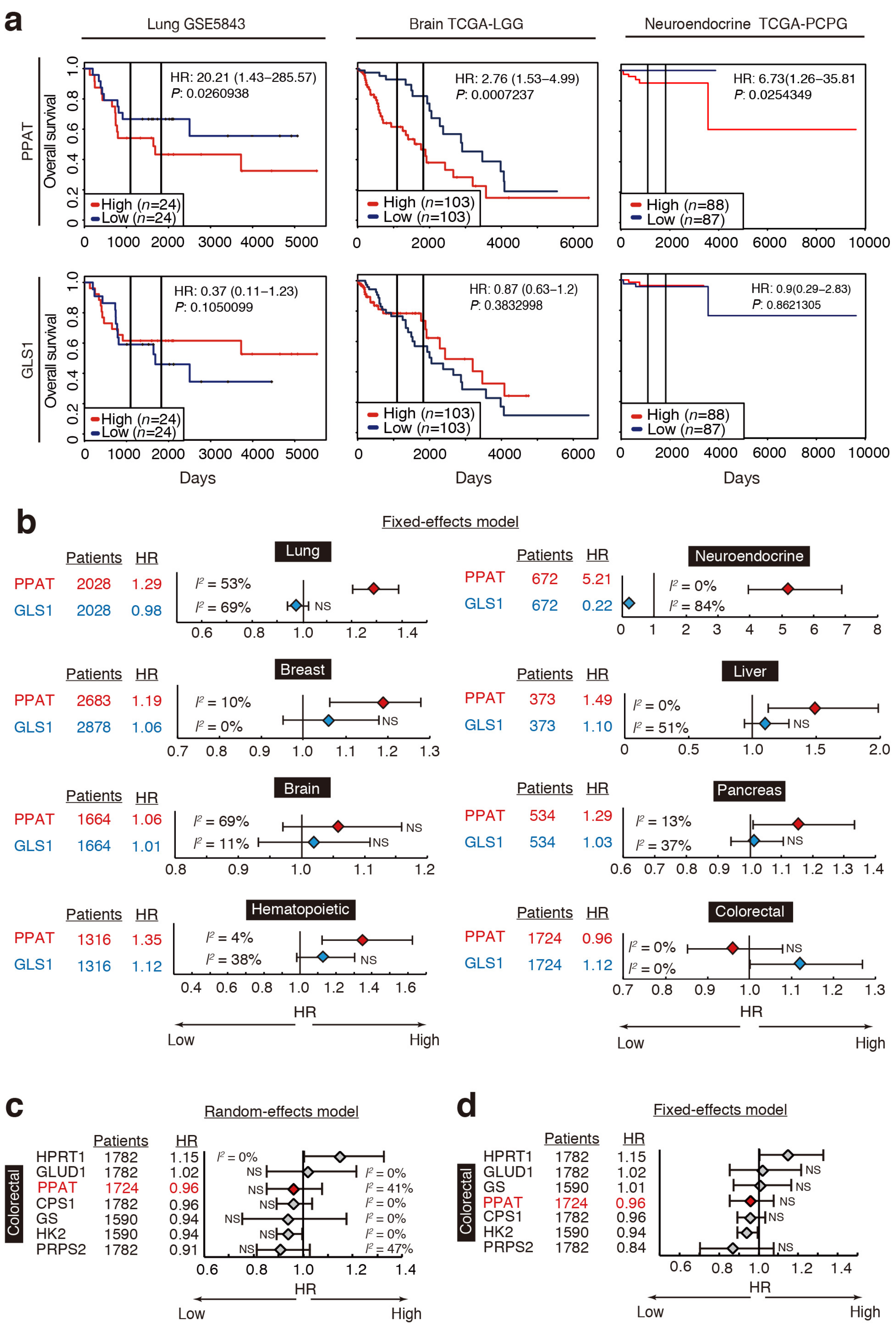

a
Lung GSE5843
Brain TCGA-LGG
Neuroendocrine TCGA-PCPG
PPAT
GLS1
Overall survival
HR: 20.21 (1.43–285.57)
P: 0.0260938
HR: 2.76 (1.53–4.99)
P: 0.0007237
HR: 6.73(1.26–35.81
P: 0.0254349
HR: 0.37 (0.11–1.23)
P: 0.1050099
HR: 0.87 (0.63–1.2)
P: 0.3832998
HR: 0.9(0.29–2.83)
P: 0.8621305
High (n=24)
Low (n=24)
High (n=103)
Low (n=103)
High (n=88)
Low (n=87)
Days
b
Fixed-effects model
Patients HR
Lung
PPAT 2028 1.29
GLS1 2028 0.98
Neuroendocrine
PPAT 672 5.21
GLS1 672 0.22
Breast
PPAT 2683 1.19
GLS1 2878 1.06
Liver
PPAT 373 1.49
GLS1 373 1.10
Brain
PPAT 1664 1.06
GLS1 1664 1.01
Pancreas
PPAT 534 1.29
GLS1 534 1.03
Hematopoietic
PPAT 1316 1.35
GLS1 1316 1.12
Colorectal
PPAT 1724 0.96
GLS1 1724 1.12
HR
Low
High
c
Random-effects model
Colorectal
Patients HR
HPRT1 1782 1.15
GLUD1 1782 1.02
PPAT 1724 0.96
CPS1 1782 0.96
GS 1590 0.94
HK2 1590 0.94
PRPS2 1782 0.91
d
Fixed-effects model
Colorectal
Patients HR
HPRT1 1782 1.15
GLUD1 1782 1.02
GS 1590 1.01
PPAT 1724 0.96
CPS1 1782 0.96
HK2 1590 0.94
PRPS2 1782 0.84

Supplementary Figure 11 | PPAT gene expression is associated with malignancy in meta-analysis with the fixed-effects model. a, Kaplan-Meier analysis of overall survival in individual cohorts of lung, brain, or neuroendocrine cancer patients from public databases. The cohorts were stratified according to median gene expression level of PPAT or GLS1. The hazard ratio (HR) and its 95% confidence interval as well as the log-rank *P* value are shown for each plot (Two-sided). Vertical black lines indicate 3- and 5-year time intervals. **b**, Cohort studies for PPAT and GLS1 gene expression and each organ in the meta-analysis were combined by means of the fixed-effects model. The integrated HR and its 95% confidence interval, the numbers of patients, and the heterogeneity score (I^2) are shown. All cohorts were divided at the median gene expression level. **c**, **d**, Meta-analysis for the expression of recently identified cancer biomarkers and PPAT in cohorts of colorectal cancer combined by means of the random-effects (**c**) or fixed-effects (**d**) models. The integrated HR and its 95% confidence interval, the numbers of patients, and the heterogeneity score (I^2) are shown. The centre of effect sizes (HR = 1.0) are shown as vertical line. Source data are provided as a Source Data file (Source Data 2).

a

Inclusion (PPAT and GLS1)

Lung

GSE5843
GSE11969
GSE26939
TCGA-LUAD
GSE11117
GSE14814
TCGA-LUSC
GSE13213
GSE17710
GSE30219
GSE3141
GSE37745
GSE19188
GSE41271
GSE50081
GSE42127

Breast

NKI
TCGA-BRCA
GSE7390
GSE3494_U133A
GS1456_U133A
GSE37751
GSE42568
GSE10893-GPL887
GSE18229-GPL887
GSE19783-GPL6480
GSE21653
GSE2607-GPL1390
GSE2607-GPL887
GSE3143
GSE48390
GSE6130-GPL1390
GSE6130-GPL887
GSE9897

Brain

GSE7696
GSE13041_U133A
GSE13041_U95v2
GSE16011
GSETCGA-GBM
TCGA-LGG
GSE16581
GSE2817
GSE30074
GSE42669
GSE4271_U133B
GSE4412_U133A

Hematopoietic

GSE12417_U133A
TCGA-AML
GSE16131_U133A
GSE22762_U133A
GSE23501
GSE2658
GSE4475

Neuroendocrine

GSE62564
TCGA-PGPC

Liver

GSE10141
TCGA-LIHC

Pancreas

GSE21501
GSE28735
TCGA-PAAD
GSE50827
GSE57495
GSE71729

Colorectal

GSE28814
GSE17536
GSE17537
TCGA-COAD
GSE16125
GSE24551
GSE28772
GSE41258
GSE29621
GSE38832
GSE39582

b

Exclusion (PPAT and GLS1)

Lung

GSE4573
GSE31210

Breast

GSE1456_U133B
GSE3494_U133B
GSE10893-GPL1390
GSE18229-GPL1290
GSE19536-GPL6480
GSE58812

Brain

GSE37418

Hematopoietic

GSE12417_U133P2
GSE22762_U133P2
GSE10846

Supplementary Figure 12 | Cancer cohort studies included in or excluded from the meta-analysis. Eighty-six cohort studies initially met the inclusion criteria and were deemed appropriate for entry into the meta-analysis. This number was reduced to 74

studies (**a**) after the subsequent exclusion of 12 studies (**b**) because some values were not clear or because of a single study being analyzed multiple times.

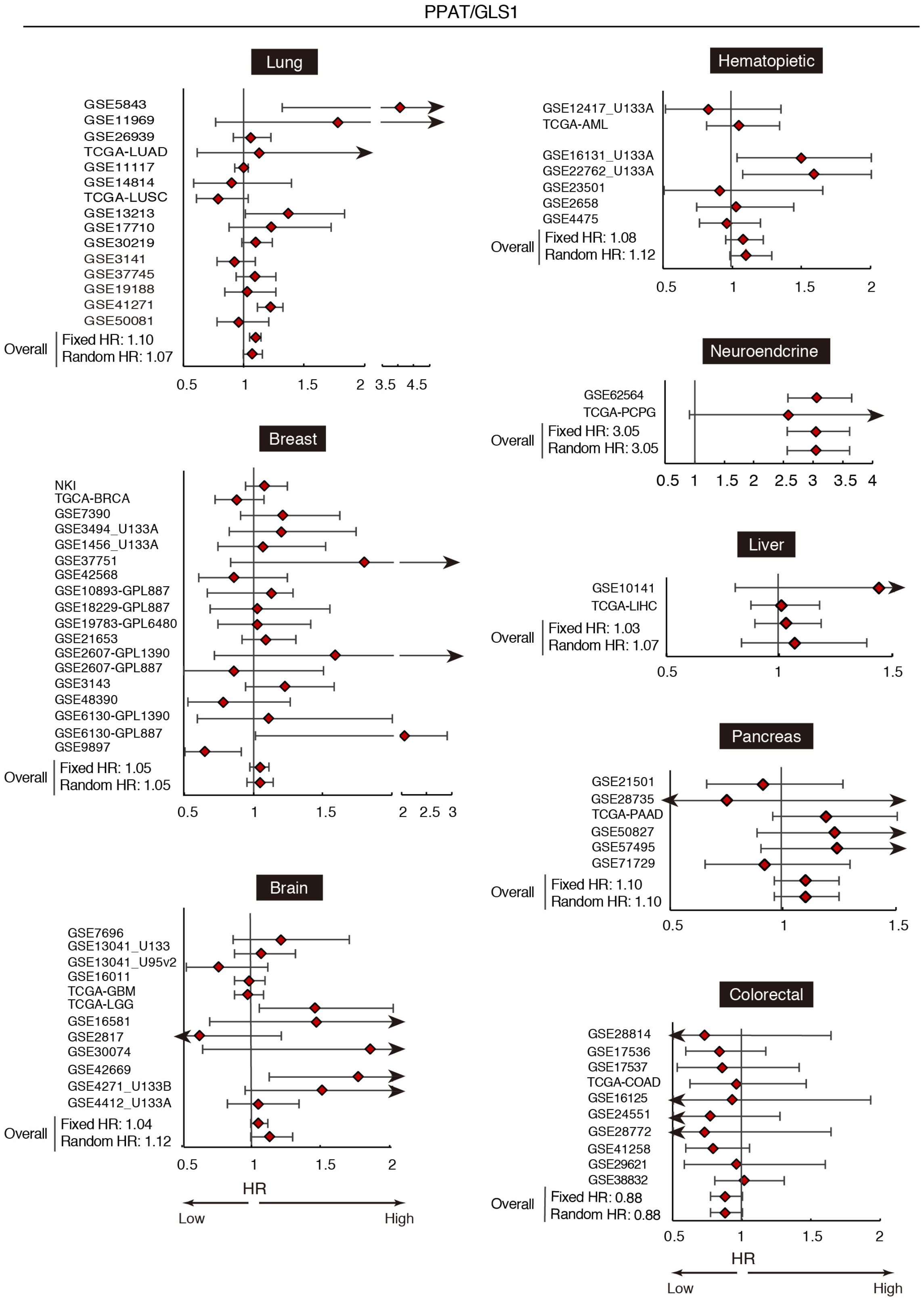
PPAT/GLS1
Lung
GSE5843
GSE11969
GSE26939
TCGA-LUAD
GSE11117
GSE14814
TCGA-LUSC
GSE13213
GSE17710
GSE30219
GSE3141
GSE37745
GSE19188
GSE41271
GSE50081
Overall
Fixed HR: 1.10
Random HR: 1.07
0.5 1 1.5 2 3.5 4.5
Breast
NKI
TGCA-BRCA
GSE7390
GSE3494_U133A
GSE1456_U133A
GSE37751
GSE42568
GSE10893-GPL887
GSE18229-GPL887
GSE19783-GPL6480
GSE21653
GSE2607-GPL1390
GSE2607-GPL887
GSE3143
GSE48390
GSE6130-GPL1390
GSE6130-GPL887
GSE9897
Overall
Fixed HR: 1.05
Random HR: 1.05
0.5 1 1.5 2 2.5 3
Brain
GSE7696
GSE13041_U133
GSE13041_U95v2
GSE16011
TCGA-GBM
TCGA-LGG
GSE16581
GSE2817
GSE30074
GSE42669
GSE4271_U133B
GSE4412_U133A
Overall
Fixed HR: 1.04
Random HR: 1.12
0.5 1 1.5 2
HR
Low
High
Hematopietic
GSE12417_U133A
TCGA-AML
GSE16131_U133A
GSE22762_U133A
GSE23501
GSE2658
GSE4475
Overall
Fixed HR: 1.08
Random HR: 1.12
0.5 1 1.5 2
Neuroendcrine
GSE62564
TCGA-PCPG
Overall
Fixed HR: 3.05
Random HR: 3.05
0.5 1 1.5 2 2.5 3 3.5 4
Liver
GSE10141
TCGA-LIHC
Overall
Fixed HR: 1.03
Random HR: 1.07
0.5 1 1.5
Pancreas
GSE21501
GSE28735
TCGA-PAAD
GSE50827
GSE57495
GSE71729
Overall
Fixed HR: 1.10
Random HR: 1.10
0.5 1 1.5
Colorectal
GSE28814
GSE17536
GSE17537
TCGA-COAD
GSE16125
GSE24551
GSE28772
GSE41258
GSE29621
GSE38832
Overall
Fixed HR: 0.88
Random HR: 0.88
0.5 1 1.5 2
HR
Low
High

Supplementary Figure 13 | Meta-analysis for the PPAT/GLS1 ratio. Cohort studies for the PPAT/GLS1 gene expression ratio and each organ in the meta-analysis were combined by means of the fixed-effects and random-effects models. The integrated hazard ratios (HRs) and its 95% confidence interval are shown. All cohorts were divided at the median gene expression ratio. Source data are provided as a Source Data file (Source Data 2).
