# Supplementary material for: A shift in glutamine nitrogen metabolism contributes to the malignant progression of cancer
Source: Nat Commun. 2020 Mar 17;11:1320. doi: 10.1038/s41467-020-15136-9 (PMC7078194; doi:10.1038/s41467-020-15136-9)
Supplement: Supplementary file 3 — Description of Additional Supplementary Files [file 41467_2020_15136_MOESM3_ESM.pdf]

**Description of Additional Supplementary Files**

File name: Supplementary Data 1

Description: Raw data for proteomics

File name: Supplementary Data 2

Description: Raw data for metabolomics
