# Supplementary material for: A shift in glutamine nitrogen metabolism contributes to the malignant progression of cancer
Source: Nat Commun. 2020 Mar 17;11:1320. doi: 10.1038/s41467-020-15136-9 (PMC7078194; doi:10.1038/s41467-020-15136-9)
Supplement: Supplementary file 6 — Reporting Summary [file 41467_2020_15136_MOESM6_ESM.pdf]

nature research

Corresponding author(s): Keiichi I. Nakayama
Last updated by author(s): Jan 14, 2020

Reporting Summary

Nature Research wishes to improve the reproducibility of the work that we publish. This form provides structure for consistency and transparency in reporting. For further information on Nature Research policies, see Authors & Referees and the Editorial Policy Checklist .

Statistics

For all statistical analyses, confirm that the following items are present in the figure legend, table legend, main text, or Methods section.

n/a	Confirmed	
☐	☒	The exact sample size (*n*) for each experimental group/condition, given as a discrete number and unit of measurement
☐	☒	A statement on whether measurements were taken from distinct samples or whether the same sample was measured repeatedly
☐	☒	The statistical test(s) used AND whether they are one- or two-sided<br>*Only common tests should be described solely by name; describe more complex techniques in the Methods section.*
☒	☐	A description of all covariates tested
☐	☒	A description of any assumptions or corrections, such as tests of normality and adjustment for multiple comparisons
☐	☒	A full description of the statistical parameters including central tendency (e.g. means) or other basic estimates (e.g. regression coefficient) AND variation (e.g. standard deviation) or associated estimates of uncertainty (e.g. confidence intervals)
☐	☒	For null hypothesis testing, the test statistic (e.g. *F*, *t*, *r*) with confidence intervals, effect sizes, degrees of freedom and *P* value noted<br>*Give P values as exact values whenever suitable.*
☒	☐	For Bayesian analysis, information on the choice of priors and Markov chain Monte Carlo settings
☐	☒	For hierarchical and complex designs, identification of the appropriate level for tests and full reporting of outcomes
☐	☒	Estimates of effect sizes (e.g. Cohen's *d*, Pearson's *r*), indicating how they were calculated

Our web collection on statistics for biologists contains articles on many of the points above.

Software and code

Policy information about availability of computer code

Data collection	MRM data analysis. Peptides for metabolic enzymes were selected from iMPAQT database and subjected to chemical synthesis (Supplementary Table1). Obtained peptides were labeled with mTRAQΔ4 reagent and spiked into mTRAQΔ0-labled sample digests. The corresponding MRM transitions with the expected retention time were obtained from iMPAQT database. MRM was performed on a triple-stage quadrupole MS instrument (QTRAP6500, SCIEX) coupled with nanoflow liquid chromatography (Eksigent nano-LC, SICEX). Endogenous peptide abundance was obtained by multiplication of the ratio of the light and heavy intensities summed for each transition and the known amount of synthetic peptide. Protein abundance was determined as the mean ± s.d. for the different peptides for each protein and replicate cultures (n = 3) for each cell line.
Data analysis	Principal component analysis was performed for the heat map derived from hierarchical clustering of measured metabolic enzymes with the use of JMP version 13. Meta-analysis was performed with the use of Review Manager 5.3. Immunoblot signals were quantified with the use of an ImageQuant LAS-4000 instrument and ImageQuant TL software TL 8.2 (GE Healthcare Life Sciences).

For manuscripts utilizing custom algorithms or software that are central to the research but not yet described in published literature, software must be made available to editors/reviewers. We strongly encourage code deposition in a community repository (e.g. GitHub). See the Nature Research guidelines for submitting code & software for further information.

Data

Policy information about availability of data

All manuscripts must include a data availability statement. This statement should provide the following information, where applicable:

- Accession codes, unique identifiers, or web links for publicly available datasets
- A list of figures that have associated raw data
- A description of any restrictions on data availability

The MS data are available under the following site.
Projects: Quantification of metabolic enzymes in TIG-3 TSM and AIG cells.

ID: PXD009543 URL: https://repository.jpostdb.org preview/130108915ad96146e3c87 Access key: 6165. Meta-analysis: Lung; GSE5843, GSE11969, GSE26939, TGCA-LUAD, GSE11117, GSE14814, TGCA-LUSC, GSE13213, GSE17710, GSE30219, GSE3141, GSE37745, GSE19188, GSE41271, GSE50081, GSE42127. Breast; NKI, TGCA-BRCA, GSE7390, GSE3494_U133A, GSE1456_U133A, GSE37751, GSE42568, GSE1893-GPL887, GSE18229-GPL887, GSE19783-GPL6480, GSE21653, GSE2607-GPL1390, GSE2607-GPL887, GSE3143, GSE48390, GSE6130-GPL1390, GSE6130-GPL887, GSE9897. Brain; GSE7696, GSE13041_U133, GSE13041_U95v2, GSE16011, TGCA-GBM, TGCA-LGG, GSE16581, GSE2817, GSE30074, GSE37418, GSE42669, GSE4271_U133B, GSE4412_U133A. NEC, GSE62564, TGCA-PGPC. Liver; GSE10141, TGCA-LIHC. Pancreas; GSE21501, GSE28735, TGCA-PAAD, GSE50827, GSE57495, GSE71729. Colorectal; GSE28814, GSE17536, GSE17537, TGCA-COAD, GSE16125, GSE24551, GSE28772, GSE41258, GSE29621, GSE38832, GSE39852

Field-specific reporting

Please select the one below that is the best fit for your research. If you are not sure, read the appropriate sections before making your selection.

☒ Life sciences ☐ Behavioural & social sciences ☐ Ecological, evolutionary & environmental sciences

For a reference copy of the document with all sections, see nature.com/documents/nr-reporting-summary-flat.pdf

Life sciences study design

All studies must disclose on these points even when the disclosure is negative.

Sample size	We searched the public database PROGgene, which compiles cohort studies from public repositories such as GEO, EBI Array Express, and The Cancer Genome Atlas (TCGA). Studies compiled through 10 March 2018 were selected for inclusion in our meta-analysis on the basis of criteria including a study duration of >7 years, with most of the selected studies having a duration of 10 to 20 years. About 80 cohort studies met the inclusion criteria and were deemed appropriate for entry into the meta-analysis form the public database PROGgene. Twelve of these studies were subsequently excluded because some values were not clear or because of a single study being analyzed multiple times, leaving a final number of ~80 cohort studies entered into the meta-analysis (Figure S6). Microarray or RNA-sequencing data for all metabolic enzymes in various organs are available online at PROGgene (http://watson.compbio.iupui.edu/chirayu/proggene/database/?url=proggene). Data were combined by means of fixed-effects or random-effects models for each organ. For integration of the HR for various organs, data were combined by means of the random-effects model.
Data exclusions	Twelve of these studies were subsequently excluded because some values were not clear or because of a single study being analyzed multiple times, leaving a final number of~80 cohort studies entered into the meta-analysis (Figure S6).
Replication	Each experiment was repeated 3-6 times independently. For details, see manuscript.
Randomization	Not applicable
Blinding	Not applicable

Reporting for specific materials, systems and methods

We require information from authors about some types of materials, experimental systems and methods used in many studies. Here, indicate whether each material, system or method listed is relevant to your study. If you are not sure if a list item applies to your research, read the appropriate section before selecting a response.

Materials & experimental systems

n/a	Involved in the study	
☐	☒	Antibodies
☐	☒	Eukaryotic cell lines
☒	☐	Palaeontology
☐	☒	Animals and other organisms
☒	☐	Human research participants
☒	☐	Clinical data

Methods

n/a	Involved in the study	
☒	☐	ChIP-seq
☐	☒	Flow cytometry
☒	☐	MRI-based neuroimaging

Antibodies

Antibodies used	Antibodies to c-Myc (ab32072) and to GLS1 (ab60709; or ab93434 in Supplementary Figs. 7e and 10a) were obtained from Abcam, those to PPAT (15401-1AP) and PDHA1 (18068-1-AP) were from Proteintech, those to Hsp90 (610419) were from BD Transduction Laboratories, and those to GAPDH (ENZ ADI-CS) were from Enzo.
Validation	The specificity of each antibody was validated by the manufacturer, and provided on the website.

Eukaryotic cell lines

Policy information about cell lines

Cell line source(s)	For retroviral infection, the mouse ecotropic retrovirus receptor was introduced into cells with the use of an amphotrop1c virus produced by HEK293T cells transfected with both pCX4-EcoVR and pGP/pE-ampho (Takara Bio). Complementary DNAs for hTERT, the SV40 early region, and human c-Myc in pCX4 were introduced into HEK293T cells together with pGP/pE-eco (Takara Bio) for the production of recombinant retroviruses. TIG-3 (Cat# JCRB0506) cells expressing the mouse ecotropic retrovirus receptor were then infected with the retrovirus encoding hTERT. The resulting cells, designated TIG-3 (T), were further infected with the retrovirus for the SV40 early region and subjected to selection to yield TIG-3 (TS) cells, which were then infected with the retrovirus for c-Myc to establish TIG-3 (TSM) cells. TIG-3, JCRB, JCRB0506; A549, ATCC, CCL185; HeLa, ATCC, CCL-2; All cancer cells in figure 5 and 7, Laboratory of Proteome Research, National Institute of Biomedical Innovation, Health, and Nutrition.
Authentication	All cell lines were authenticated by the providers.
Mycoplasma contamination	Cells were tested for mycoplasma using the MC-210 (KAC, Cat #88101-2) and PCR analysis. All cells were confirmed to be free of mycoplasma.
Commonly misidentified lines (See ICLAC register)	Not applicable

Animals and other organisms

Policy information about studies involving animals; ARRIVE guidelines recommended for reporting animal research

Laboratory animals	Six-week-old female athymic nude (Balb/c-nu/nu) mice were obtained from CLEA Japan.
Wild animals	No wild animals were used.
Field-collected samples	No field collected samples were used in the study.
Ethics oversight	All mouse experiments were approved by the Animal Ethics Committee of Kyushu University.

Note that full information on the approval of the study protocol must also be provided in the manuscript.
